# Protective Effects of Ethanolic Extract from Rhizome of *Polygoni avicularis* against Renal Fibrosis and Inflammation in a Diabetic Nephropathy Model

**DOI:** 10.3390/ijms22137230

**Published:** 2021-07-05

**Authors:** Jung-Joo Yoon, Ji-Hun Park, Yun-Jung Lee, Hye-Yoom Kim, Byung-Hyuk Han, Hong-Guang Jin, Dae-Gill Kang, Ho-Sub Lee

**Affiliations:** 1 Hanbang Cardio-Renal Syndrome Research Center, Wonkwang University, Jeollabuk-do, Iksan 54538, Korea; mora16@naver.com (J.-J.Y.); jihuncjstk@naver.com (J.-H.P.); shrons@wku.ac.kr (Y.-J.L.); hyeyoomc@naver.com (H.-Y.K.); arum0924@nate.com (B.-H.H.); 2 College of Oriental Medicine and Professional Graduate School of Oriental Medicine, Wonkwang University, Jeollabuk-do, Iksan 54538, Korea; 3 Institute of Pharmaceutical Research and Development, College of Pharmacy, Wonkwang University, Jeollabuk-do, Iksan 54538, Korea; hg_jin1979@163.com; 4 School of Pharmacy and Life Sciences, Jiujiang University, Jiujiang 332005, China

**Keywords:** diabetic nephropathy, *Polygoni avicularis*, db/db mice, renal dysfunction, inflammation

## Abstract

Progressive diabetic nephropathy (DN) in diabetes leads to major morbidity and mortality. The major pathological alterations of DN include mesangial expansion, extracellular matrix alterations, tubulointerstitial fibrosis, and glomerular sclerosis. *Polygoni avicularis* is widely used in traditional oriental medicine and has long been used as a diuretic, astringent, insecticide and antihypertensive. However, to the best of the authors’ knowledge, the effects of the ethanolic extract from rhizome of *Polygoni avicularis* (ER-PA) on DN have not yet been assessed. The present study aimed to identify the effect of ER-PA on renal dysfunction, which has been implicated in DN in human renal mesangial cells and db/db mice and investigate its mechanism of action. The in vivo experiment was performed using *Polygoni avicularis*-ethanol soluble fraction (ER-PA) and was administrated to db/db mice at 10 and 50 mg/kg dose. For the in vitro experiments, the human renal mesangial cells were induced by high glucose (HG, 25 mM). The ER-PA group showed significant amelioration in oral glucose tolerance, and insulin resistance index. ER-PA significantly improved the albumin excretion and markedly reduced plasma creatinine, kidney injury molecule-1 and C-reactive protein. In addition, ER-PA significantly suppressed inflammatory cytokines. Histopathologically, ER-PA attenuated glomerular expansion and tubular fibrosis in db/db mice. Furthermore, ER-PA suppressed the expression of renal fibrosis biomarkers (TGF and Collagen IV). ER-PA also reduced the NLR family pyrin domain containing 3 inflammatory factor level. These results suggest that ER-PA has a protective effect against renal dysfunction through improved insulin resistance as well as the inhibition of nephritis and fibrosis in DN.

## 1. Introduction

Diabetic nephropathy is a typical kidney disease that occurs as result of diabetes mellitus [1]. Diabetic renal damage, one of the most common complications of diabetes [2], is the most frequent cause of end-stage renal failure [3]. In diabetes, kidney damage affects various structures of the kidneys and is mainly characterized by an increased deposition of the extracellular matrix (ECM), aggravated glomerular fibrosis, and the overexpression of chemokines, leading to tubulointerstitial damage [4,5]. The consequences involve kidney fibrosis, proteinuria, and kidney inflammation. Diabetic nephropathy is one of the leading causes of diabetes mellitus progressing to end-stage renal disease and is the most common cause of kidney failure [6].

TGF-β is a key regulator of fibrosis by promoting the accumulation of ECM. TGF-β induces phosphorylation and activation of Smad signaling pathway [7]. In particular, r-Smad serves a pivotal role in controlling the growth and differentiation of cells involved in intracellular signaling of the TGF-β1 superfamily [8]. The depletion of nephrin and podocin proteins in podocytes following glomerular injury causes severe proteinuria [9]. 

The activation of NF-κB may serve an important role in the pathogenesis of DN [10]. Mononuclear cell invasion and abnormal expression of inflammatory mediators, including intercellular adhesion molecule-1 (ICAM-1), monocyte chemoattractant protein-1 (MCP-1), and TGF-β1 are observed in renal tissues at early stages of DN [11]. TGF-β1 is considered to serve an important role in mediating fibrosis in DN [12]. Previous studies have suggested that TGF-β1 mediates the accumulation of ECM in mesangial and tubular cells and that the inhibition of TGF-β1 signaling significantly reduces renal fibrosis and decreases the mRNA levels of major mediators of ECM deposition in db/db mice [13]. Ligand binding causes phosphorylation of the Smad-2 and Smad-3 proteins. Activated r-Smad acts as a co-Smad (Smad-4) and mainly forms a trans-β-active complex, which is transported into the nucleus to modulate the expression of the target genes, including TGF-β [14]. Connective tissue growth factor (CTGF) may also contribute to diabetic renal disease through the inhibition of matrix decomposition and the induction of ECM synthesis [15]. In DN, ECM degradation is reduced and CTGF suppresses the decomposition of human renal cell substrates [16]. The NLR family pyrin domain containing 3 (NLRP3) undergoes oligomerization in the presence of the adaptor protein apoptosis-associated speck-like protein (ASC) and protease caspase-1 to form a protein complex, termed as the inflammasome. The formation of inflammasome is important for the auto-processing of caspase-1 and activation of cytokines, pro-IL-1b, and pro-IL-18 [17].

Herbs have been used for thousands of years, and more recently, herbal remedies are being considered as complementary medicines for disease prevention, making it a notable treatment option for the treatment of the disease [18,19,20]. *Polygonum aviculare* L. (PA), a member of the Polygonaceae family, is used as a traditional medicine. PA is a safe and effective diuretic herb [21] that has multiple biological effects including antioxidant [22], antitumor [23], and anti-inflammatory activities [24]. PA extract has a high content of phenolics and flavonoids, which show DNA-protective activities [22,25]. However, the effect of PA on DN has yet to be elucidated. Therefore, the purpose of the present study was to investigate whether PA extract has preventive effects on diabetic nephropathy, which is associated with renal dysfunction in db/db mice, an animal model of type 2 diabetes.

## 2. Materials and Methods

### 2.1. Preparation of ER-PA

The dried rhizome of harvesting one-year-old plants (*Polygoni avicularis*) (1.0 kg), which was purchased from Daehak Hanyakguk (a dispensary of Oriental medicine), Iksan, Korea, was cut and then extracted with ethanol (10 L) for 3 h at 80 °C. The extract was filtered using a filter paper and the filtrate was concentrated under reduced pressure to obtain 126 g of extract. The extract was suspended in distilled water and treated with 1.5 L of ethyl acetate. The fractions were partitioned in a separatory funnel to obtain 17.85 g of ethyl acetate fraction.

### 2.2. Isolation of Compounds from ER-PA

**Figure 1:**
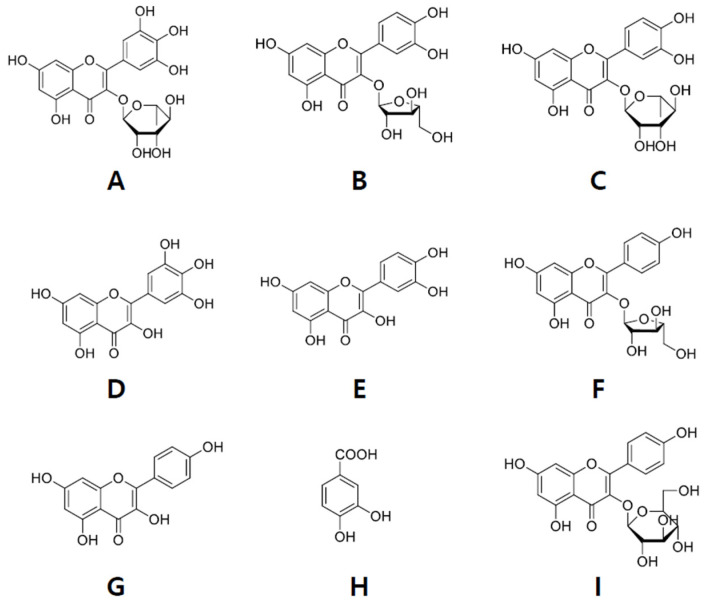
Chemical structure of compounds isolated from Polygonum aviculare L. (**A**) Myricetin-3-O-rhamnoside; (**B**) Quercetin-3-O-arabinofuranoside; (**C**) Quercetin-3-O-rhamnoside; (**D**) Myricetin; (**E**) Quercetin; (**F**) Kaempferol-3-O-arabinofuranoside; (**G**) Kaempferol; (**H**) Protocatechuic acid; (**I**) Kaempferol-3-O-β-D-glucopyranoside.

The ethanol-soluble fraction (2.78 g) was subjected to column chromatography (CC) over a Sephadex LH-20 column using a chloroform (CHCl_3_):methanol (MeOH) = 1:1 gradient system. The fractions were combined based on their thin-layer chromatography (TLC) patterns to yield subfractions, designated as E1–E7. Fraction E4 (838.1 mg) was subjected to medium-pressure liquid chromatography (MPLC; ODS-S-50-B, 26 × 300 mm) using H_2_O:MeOH = 80:20 → 0:100 to obtain nine fractions (E41–H49). Fraction E43 (93.9 mg) was further subjected to silica gel CC using a dichloromethane (CH_2_Cl_2_):MeOH = 8:1 → 2:1 gradient system to obtain six subfractions (E431–E436). Subfraction E433 was then purified with reverse-phase high-performance liquid chromatography (RP-HPLC; YMC-Pack C18, 20 × 150 mm) using H_2_O:MeOH = 50:50 → 35:65 for 30 min and then by normal phase (NP)-HPLC (YMC-Pack SIL-06, 10 × 250 mm) using H_2_O:EtOH = 75:25 → 40:60 for 30 min to obtain myricetin-3-O-rhamnoside [26] (5.6 mg, 0.20%, Figure 1A). Fraction E44 (76.1 mg) was subjected to silica gel CC and eluted using a hexane:acetone = 2:1 → 1:3 gradient system to obtain quercetin [27] (8.8 mg, 0.32%, Figure 1F). Fraction E45 (103.8 mg) was subjected to Sephadex LH-20 CC (CHCl_3_:hexane:MeOH = 5:3:1) to obtain four subfractions (E451–E454). Subfraction E452 was then purified by MPLC (ODS-S-50-A, 11 × 300 mM) using H_2_O:MeOH = 60:40 → 40:60 and finally by NP HPLC (YMC-Pack SIL-06, 10 × 250 mm) using a H_2_O:EtOH = 80:20 → 70:30 elution system for 30 min to separate quercetin-3-O-arabinofuranoside [28] (8.3 mg, 0.30%, Figure 1B), quercetin-3-O-rhamnoside [29] (6.0 mg, Figure 1C) and protocatechuic acid [30] (2.0 mg, 0.07%, Figure 1I). Fractions E46 (14.4 mg) and E47 (34.9 mg) were subjected to silica gel CC using a CHCl_3_:MeOH = 8:1 → 6:1 gradient system to obtain six subfractions (E4671–E4676). Subfraction E4674 was later purified by NP HPLC (YMC-Pack SIL-06, 10 × 250 mM) using a H_2_O:EtOH = 82:18 → 75:25 system for 30 min to obtain kaempferol-3-O-arabinofuranoside [27] (22.3 mg, 0.80%, Figure 1G) and kaempferol-3-O-β-D-glucopyranoside [27] (7.2 mg, 0.26%). Fraction E49 (16.2 mg) was subjected to RP-HPLC (YMC-Pack C18, 20 × 150 mM) first using H_2_O:MeOH = 30:70 → 10:90 for 30 min and then using hexane:acetone = 2:1 → 1:3 in a gradient system to obtain kaempferol [28] (3.3 mg, 0.12%, Figure 1H). Fraction E7 (51.4 mg) was subjected to MPLC (ODS-S-50-A, 11 × 300 mM) with H_2_O:MeOH = 50:50 → 0:100 to obtain myricetin [31] (5.6 mg, 0.20%, Figure 1E). 

### 2.3. Experimental Animals and Diets

All experimental procedures were performed in accordance with the National Institute of Health Guide for the Care and Use of Laboratory Animals and were approved by the Institutional Animal Care and Utilization Committee for Medical Science of Wonkwang University (approval number: WKU 14-50). In brief, 12-week-old male db/db mice (C57BL/6J Lepr) and age-matched non-diabetic db/m mice (C57BLKS/J) were purchased from CLEA Japan, Inc. After one week of acclimation, the mice were randomly divided into five groups (*n* = 8 per group; total 40) as follows: Control group comprising db/m mice (db/m); negative control group comprising db/db mice (db/db); positive control group comprising db/db mice daily treated with 20 mg/kg aminoguanidine (AG); db/db mice daily treated with a low concentration (10 mg/kg) of ER-PA (PAL); and db/db mice treated daily with a high concentration (50 mg/kg) of ER-PA (PAH). The mice were subjected to diabetes through genetic modification. Any animal that died or was severely injured during the experiment was excluded from study. The primary outcome of the present study involved assessing changes in biomarkers that improved the DN. The primary indicators included changes in blood glucose and insulin indicators. The secondary evaluation variables were changes in renal morphology and nephrin expression to measure improvements in renal functions. Renal fibrosis and inflammation were detected as changes in the expression of related factors. The mice were housed in a room automatically maintained at a temperature of 23 ± 2 °C, humidity of 50–60% and a 12-h light/dark cycle throughout the experiment. Body weight and water/food consumption were measured weekly. They were anesthetized with 4% isoflurane using an Anesthesia Tabletop Bracket with a N_2_O and O_2_ Flow Meter System (Small Animal Ventilator; Harvard Apparatus) and were sacrificed by incision of the abdominal artery.

### 2.4. Cell Cultures

Primary human renal mesangial cells were purchased from ScienCell Research Laboratories, Inc. and cultured in a low-glucose Dulbecco’s modified Eagle’s medium (DMEM; Gibco; Thermo Fisher Scientific, Inc., Waltham, MA, USA) supplemented with 10% fetal bovine serum (Gibco; Thermo Fisher Scientific, Inc.) and 1% antibiotic-antimycotic (Gibco; Thermo Fisher Scientific, Inc.). The dispersed mesangial cells were incubated in a humidified incubator at 37 °C under 95% air and 5% CO_2_.

### 2.5. Estimation of Blood Glucose and the Oral Glucose Tolerance Test (OGTT)

The concentration of glucose in blood was measured every two weeks using blood samples obtained from the tail vein, with a glucometer (OneTouch Ultra) and Test Strip (Life Scan Inc., Milpitas, CA, USA). The OGTT was performed two days apart at eight weeks. For the OGTT, basal blood glucose concentrations were measured after 10–12 h of overnight fasting. A glucose solution (2 g/kg body weight) was immediately administered via oral gavage and blood samples were obtained after 30, 60, 90, and 120 min.

### 2.6. Analysis of Plasma Biochemical Markers

Insulin, C-reactive protein (CRP), kidney injury molecule-1 (KIM-1), and hemoglobin A1c (HbA1c) levels in the plasma were measured with ELISA using a mouse insulin ELISA kit (AKRIN-011T, Shibayagi Co., Ltd., Gunma Prefecture, Japan), commercial mouse CRP ELISA kit (LS-F4264, LSBIO, Ltd., Seattle, WA, USA), commercial KIM-1 ELISA kit (LS-F24859, LSBIO, Ltd.) and commercial HbA1c ELISA kit (MBS776343, MyBioSource, Inc., San Diego, CA, USA), respectively.

### 2.7. Monitoring Renal Function

Mice from each group were maintained in separate metabolic cages for two days, to collect urine and measure water and food intake. Urine samples were used to determine creatinine level, osmolality and other parameters related to renal function. The levels of creatinine in the plasma were colorimetrically measured using a spectrophotometer (Milton Roy). The concentrations of ions were measured using an electrolyte analyzer (NOVA 5; Nova Biomedical) and osmolality was determined using an Advanced CRYOMATIC osmometer (model 3900; Advanced Instruments, LLC., Norwood, MA, USA).

### 2.8. Immunohistochemical and Morphological Staining of Kidney Tissue

For immunohistochemical and morphological analyses, isolated kidney tissues were fixed in 4% paraformaldehyde for 48 h at 4 °C, then incubated with 30% sucrose for two days. Each tissue was embedded in an embedding medium, optimum cutting temperature (O.C.T.) compound (Sakura Finetek USA, Torrance, CA, USA), frozen in liquid nitrogen, and stored at −70 °C until analysis. Frozen sections for immunohistochemical staining were cut to a thickness of 10 μm, with a Shandon Cryotome SME (Thermo Fisher Scientific, Inc.) and placed on poly L-lysine-coated slides (Thermo Fisher Scientific, Inc.). The slides were air dried overnight at room temperature and stored at −70 °C until immunostaining. Slides were immunostained using Histostain^®^-SP kits, as per the labeled-[strept] avidin-biotin (LAB-SA) method (Invitrogen; Thermo Fisher Scientific, Inc.). For quantitative analysis, the average score of 10–20 randomly selected areas was calculated using the NIH Image analysis software, ImageJ (National Institutes of Health, version 1.49). The age of 16 weeks, mice were anesthetized and perfused with ice-cold Ringer solution before being perfused and fixed with 10% (*v*/*v*) buffered formalin in 50 mM potassium phosphate buffer (pH 7.0) for 48 h at 4 °C. For morphometric analysis, the kidney was removed and embedded in paraffin to prepare 4-μm tissue slices. The tissue slices were stained with periodic acid-Schiff (PAS) and images were captured and analyzed using ImageJ.

### 2.9. Western Blot Analysis of Kidney Samples

Kidney homogenates were prepared in an ice-cold buffer containing 250 mM sucrose, 1 mM ethylenediaminetetraacetic acid, 0.1 mM phenylmethylsulfonyl fluoride and 20 mM potassium phosphate buffer (pH 7.6). The homogenates were centrifuged at 8000 rpm for 10 min at 4 °C and the supernatant obtained was further centrifuged at 13,000 rpm for 5 min at 4 °C and used as the cytosolic fraction for protein analysis. The protein concentrations were determined using a Bradford protein assay [32]. The recovered proteins (40 μg) were separated on 10% sodium dodecyl sulfate-polyacrylamide gels and transferred onto nitrocellulose membranes. Membranes were blocked with 5% bovine serum albumin in 0.05% Tween 20 Tris-buffered saline (TBS-T) for 1 h in room temperature. The blots were then incubated with antibodies against TGF-β1(sc-65378), Smad-2 (sc-6200), Smad-3 (SC-101154), Smad-4 (SC-7966), CTGF (SC-373936), nephrin (SC-32530), ICAM-1 (SC-8439), MCP-1 (SC-52701), ASC (SC-514414), and caspase-1 (SC-56036) (Santa Cruz Biotechnology, Inc., Dallas, TX, USA); Collagen IV (ab227616) and NLRP3 (ab263899) (Abcam (Cambridge, UK), overnight at 4 °C. It was cultured by diluting it with an antibody at a ratio of 1:1000. The blots were washed several times with TBS-T and probed with a horseradish peroxidase-conjugated secondary antibody for 1 h. The immunoreactive bands were visualized using an enhanced chemiluminescence substrate (Amersham; Cytiva) and densitometrically analyzed using a Chemi-doc image analyzer (Bio-Rad Laboratories, Inc., Hercules, CA, USA).

### 2.10. Reverse Transcription-Quantitative (RT-q) PCR

Cell were collected (5 × 10^4^) and a kit from Qiagen (RNeasy™ Plus mini kit) was used for RNA isolation from cell cultures and the RNA quality was assessed from the ratio of absorbance measured at 260/280 nm, using a UV-spectrophotometer. All experimental procedures were performed according to the manufacturer’s protocol. RT-qPCR analysis was performed in a 96-well plate using the Opticon MJ Research instrument (Bio-Rad Laboratories, Inc.) and an optimized standard SYBR Green 2-step RT-qPCR kit protocol (DyNAmo™, Finnzymes; Thermo Fisher Scientific, Inc.). The specific sense and antisense primers were as follows: ICAM-1, 5′-GCT GCT ACC ACA CTG ATG ACG ACA A-3′ (sense) and 5′-CAG TGA CCA TCT ACA GCT TTC CGG-3′ (anti-sense); MCP-1, 5′-GATCTCAGTGCAGAGGCTCG-3′ (sense) and 5′-TGC TTG TCC AGG TGG TCC AT-3′ (anti-sense); Collagen IV, 5′-GGT GTT GCA GGA GTG CCA G-3′ (sense) and 5′-GCA AGT CGA AAT AAA ACT CAC CAG-3′ (anti-sense); CTGF, 5′-GCA AAT AGC CTG TCA ATC TC-3′ (sense) and 5′-TCC ATA AAA ATC TGG CTT GT-3′ (anti-sense); TGF-β1, 5′-CAA CAA TTC CTG GCG TTA CCT TGG-3′ (sense) and 5′-GAA AGC CCT GTA TTC CGT CTC CTT-3′ (anti-sense); NLRP-3, 5′-CTG GAG ATC CTA GGT TTC TCT G-3′ (sense) and 5′-CAG GAT CTC ATT CTC TTG GAT C-3 (anti-sense); ASC, 5′-ATC CAG GCC CCT CCT CAGT-3′ (sense) and 5′-GTT TGT GAC CCT CGC GAT AAG-3′ (anti-sense); GAPDH, 5′-CGA GAA TGG GAA GCT TGT CAT C-3′ (sense) and 5′-CGG CCT CAC CCC ATT TG-3′ (anti-sense). The PCR data were the result of repeating 3 times and analyzed using the software provided by the manufacturer Monitor™ analysis software (#CFB-3120EDU). 

### 2.11. Statistical Analysis

Values are shown as the mean ± standard error (S.E.), and the data were analyzed using Sigma Plot 10.0 software (SPSS Inc., Chicago, IL, USA) to compare mean values between groups in a one-way ANOVA followed by a Dunnett’s test or Student’s *t*-test. A value of *p* < 0.05 was considered statistically significant. Thirty-two db/db mice were randomly divided into groups and used for the study, with no exceptions except unexplained deaths (the cause of death for one animal: could not be identified by autopsy) and deep wounds due to quarrels (two animals).

## 3. Results

### 3.1. Effects of the Ethanolic Extract from Rhizome of PA on Fluid Metabolism

**Figure 2:**
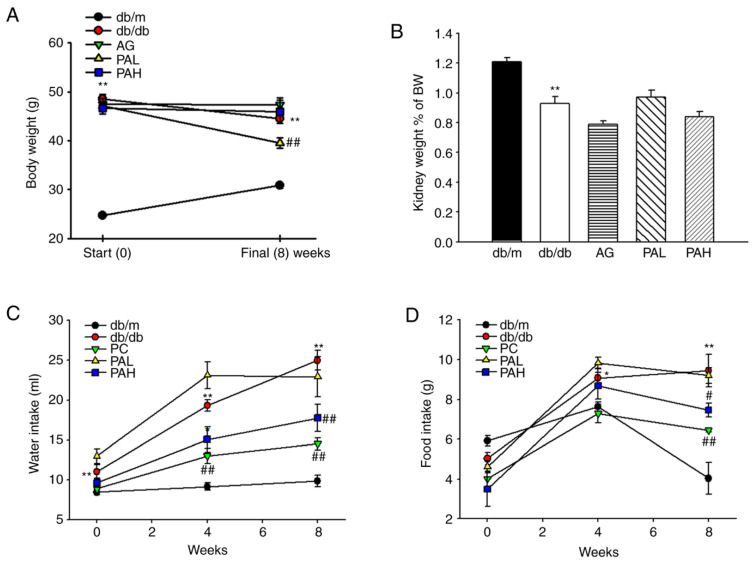
Effects of ER-PA on (**A**) body weight; (**B**) kidney weight; (**C**) food intake and (**D**) water intake in db/db mice. Data are presented as mean ± standard deviation (*n* = 8). * *p* < 0.05, ** *p* < 0.01 vs. db/m; # *p* < 0.05, ## *p* < 0.01 vs. db/db. PA, *Polygoni avicularis*; BW, body weight.

As shown in Figure 2A, all db/db mice groups showed a significantly higher body weight throughout the experiment than the normal group (db/m). However, the PAL group showed a significant decrease at 8 weeks (*p* < 0.01). As a result of evaluating the change in kidney weight (kidney weight as % of body weight), the kidney weight in the db/db group was lower compared with that in the db/m group and no difference was observed following ER-PA administration (Figure 2B). Food and water intake were significantly higher in the db/db group compared with the db/m group. However, food and water intake levels in the PAH group showed a significant decrease compared with the db/db group at eight weeks, which was similarly to that of the AG group (Figure 2C,D).

### 3.2. Effects of the Ethanolic Extract from Rhizome of PA on Glucose Tolerance and Insulin Resistance

**Figure 3:**
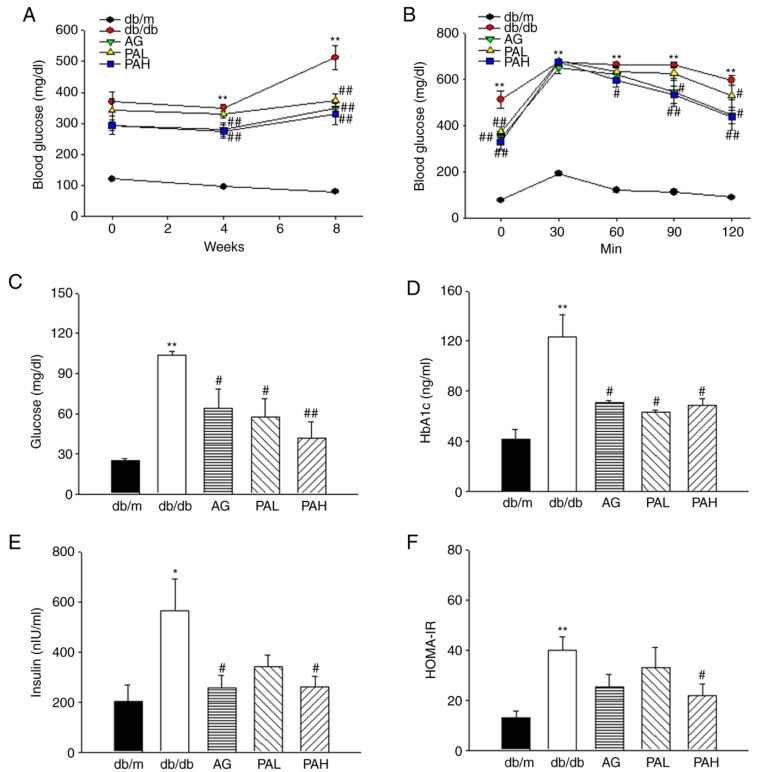
Effects of ER-PA on blood glucose (**A**); oral glucose tolerance test (**B**); glucose (**C**); HbA1c (**D**); insulin (**E**) and HOMA-IR (**F**). Values are expressed as mean ± standard error of the mean (*n* = 8). * *p* < 0.05, ** *p* < 0.01 vs. db/m; # *p* < 0.05, ## *p* < 0.01 vs. db/db. PA, *Polygoni avicularis*; HbA1c, hemoglobin A1c; HOMA-IR, insulin resistance index; AG, db/db mice daily treated with 20 mg/kg aminoguanidine; PAL, db/db mice daily treated with a low concentration (10 mg/kg) of ER-PA; PAH, db/db mice treated daily with a high concentration (50 mg/kg) of ER-PA.

To understand the effects of ER-PA on glucose metabolism and insulin resistance in db/db mice, fasting blood glucose, glucose tolerance, and insulin levels were measured. Blood glucose levels in all db/db mice groups throughout the experiment were significantly higher compared with the db/m group. However, at eight weeks, the AG, PAL, and PAH groups showed significantly lower blood glucose levels compared with the db/db group (Figure 3A, *p* < 0.01). These results were similar in the results measured through biochemical analysis from blood collected after the experiment was completed (Figure 3C). OGTT was performed to determine the effect of ER-PA on glucose tolerance in db/db mice. The blood glucose concentration of OGTT markedly increased in db/db group, whereas the AG, PAL, and PAH groups exhibited significantly suppressed blood glucose concentration 60, 90, and 120 min following the glucose load (Figure 3B; *p* < 0.05; *p* < 0.05; *p* < 0.01). Correspondingly, HbA1c levels were significantly decreased in AG, PAL, and PAH groups compared with db/db group at the end of treatment (Figure 3D, *p* < 0.05). 

As shown in Figure 3E, plasma insulin levels were markedly higher in the db/db group (563.30 ± 127.05) compared with the db/m group (204.35 ± 63.62). However, AG and PAH groups exhibited significantly lowered plasma insulin levels compared with the db/db group (*p* < 0.05). Additionally, the insulin resistance index (HOMA-IR) values were significantly lower in the PAH group compared with the db/db group (Figure 3F, *p* < 0.05, 21.95 ± 4.73 vs 40.00 ± 5.26). The results demonstrated that the high dose of ER-PA, 50 mg/kg, was the most effective in decreasing blood glucose levels and improving insulin resistance.

### 3.3. Effects of the Ethanolic Extract from Rhizome of PA on Renal Function and Glomerular Morphological Changes

**Figure 4:**
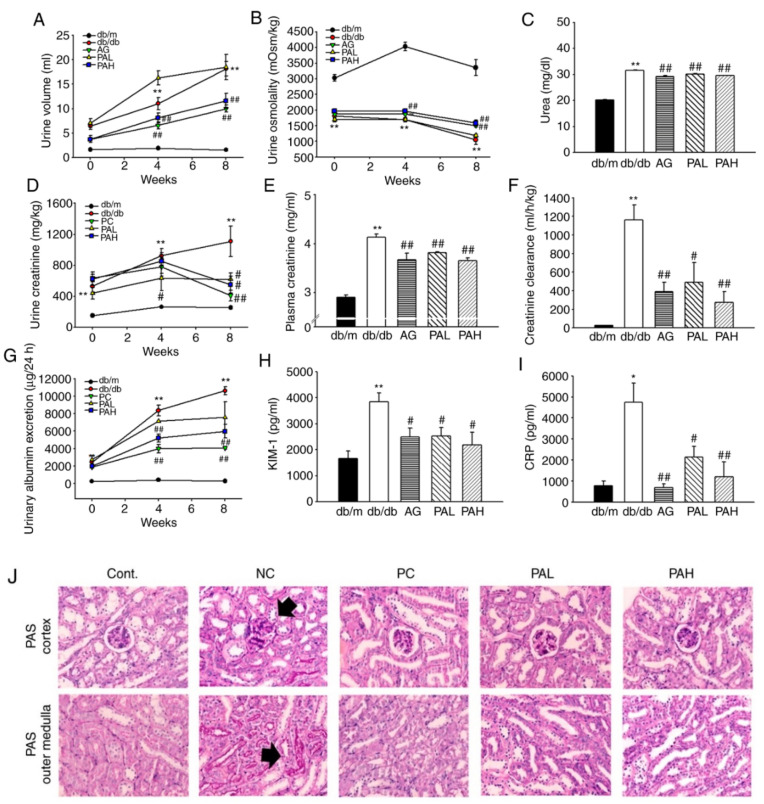
Effects of ER-PA on renal function and glomerular morphological changes. Effect of ER-PA on urine volume (**A**); urine osmolality (**B**); Urea (**C**); urine creatinine (**D**); plasma creatinine (**E**); creatinine clearance (**F**); urinary albumin excretion (**G**); KIM-1 (**H**) and CRP (**I**). Values are expressed as mean ± standard error of the mean (*n* = 8). * *p* < 0.05, ** *p* < 0.01 vs. db/m; # *p* < 0.05, ## *p* < 0.01 vs. db/db. (**J**) Representative microscopic photographs of a kidney stained with PAS. Kidney sections in cortex (glomerulus) and outer medulla obtained from the db/m group, db/db group, AG group, PAL group and PAH group (*n* ≥ 3; magnification, ×200). PA, *Polygoni avicularis*; AG, db/db mice daily treated with 20 mg/kg aminoguanidine; PAL, db/db mice daily treated with a low concentration (10 mg/kg) of ER-PA; PAH, db/db mice treated daily with a high concentration (50 mg/kg) of ER-PA.

The urine volume was significantly higher for the db/db group compared with the db/m group during the experiment period (weeks 0–8). However, the urine volume in the AG and PAH groups was significantly lower compared with the db/db group during the entire experimental period (Figure 4A; *p* < 0.01). Urine osmolality was higher in the PAH group compared with db/db mice (Figure 4B; 1491.75 ± 94.25 vs. 1275.2 ± 139.09; *p* < 0.01), similarly to levels observed in AG group. In addition, after 8 weeks, the urine urea concentration significantly reduced in the AG, PAL and PAH groups compared with that in the db/db group (*p* < 0.01; Figure 4C). The urinary and plasma creatinine levels in the db/db group were markedly higher compared with the db/m group (*p* < 0.01). Creatinine levels were lower in PAL and PAH groups compared with the db/db group (Figure 4D; 619.2 ± 84.84 and 551.7 ± 110.87 vs. 1108.49 ± 196.2; Figure 4E, 3.82 ± 0.02 and 3.65 ± 0.06 vs. 4.13 ± 0.07). The creatinine clearance rate in the AG, PAL, and PAH groups also significantly improved compared with that in the db/db group (Figure 4F, *p* < 0.01, *p* < 0.05, *p* < 0.01). The urinary excretion of sodium, chloride and potassium was significantly lower in PAL and PAH groups compared with the db/db group (data not shown). As shown in Figure 4G, compared with db/m group, albuminuria was established in db/db group at the initial treatment and gradually increased. After 4 weeks, urinary albumin excretion was significantly reduced in the AG and PAH groups, the effect continued through 8 weeks (*p* < 0.01). Furthermore, KIM-1 (an early biomarker of acute kidney injury) and CRP (a biomarker of inflammation) were significantly reduced in the AG, PAL and PAH groups (Figure 4H,I). To determine the effect of ER-PA on the kidney structure, particularly the glomerulus, kidney cross-sections were stained with periodic acid Schiff (PAS). PAS staining revealed glomerular basement membrane thickening and mesangial expansion, as well as increased accumulation of ECM in db/db group. However, the PAL and PAH treatment ameliorated mesangial expansion, similarly to levels observed in the AG group (Figure 4J).

### 3.4. Effects of the Ethanolic Extract from Rhizome of PA on Nephrin Levels

**Figure 5:**
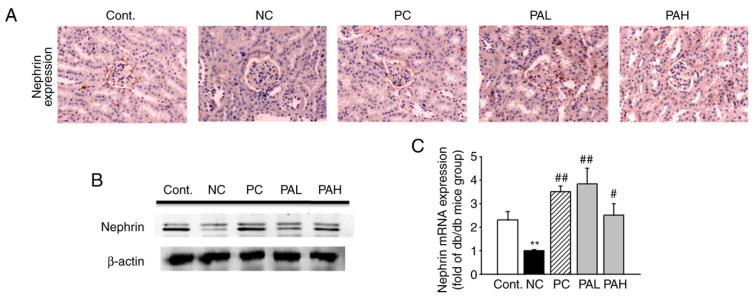
Effect of ER-PA on nephrin immunoreactivity in db/db mice. Expression of nephrin in kidneys was determined with (**A**) immunohistochemistry staining; (**B**) western blotting and (**C**) reverse transcription-quantitative PCR analysis. Values are expressed as mean ± standard error of the mean (*n* = 8). ** *p* < 0.01 vs. db/m; # *p* < 0.05, ## *p* < 0.01 vs. db/db (*n* ≥ 3; magnification, ×200). PA, *Polygoni avicularis*; AG, db/db mice daily treated with 20 mg/kg aminoguanidine; PAL, db/db mice daily treated with a low concentration (10 mg/kg) of ER-PA; PAH, db/db mice treated daily with a high concentration (50 mg/kg) of ER-PA.

As shown in Figure 5A, to determine whether ER-PA ameliorates early glomerular injury by the loss of glomerular nephrin expression, immunohistochemistry was performed. The staining of nephrin in the db/m group was greater than the db/db group. However, in the PAL and PAH groups, the expression of nephrin was significantly increased compared with db/db mice. To confirm the results obtained from immunohistochemistry, Western blot analysis and RT-qPCR were performed. The nephrin protein and mRNA level significantly increased in the PAL and PAH groups compared with the db/db group (Figure 5B,C; *p* < 0.01; *p* < 0.05). These results suggested that PAH can improve kidney damage by upregulating the expression of nephrin, a biomarker of early glomerular damage.

### 3.5. Effect of the Ethanolic Extract from Rhizome of PA on Renal Fibrosis

**Figure 6:**
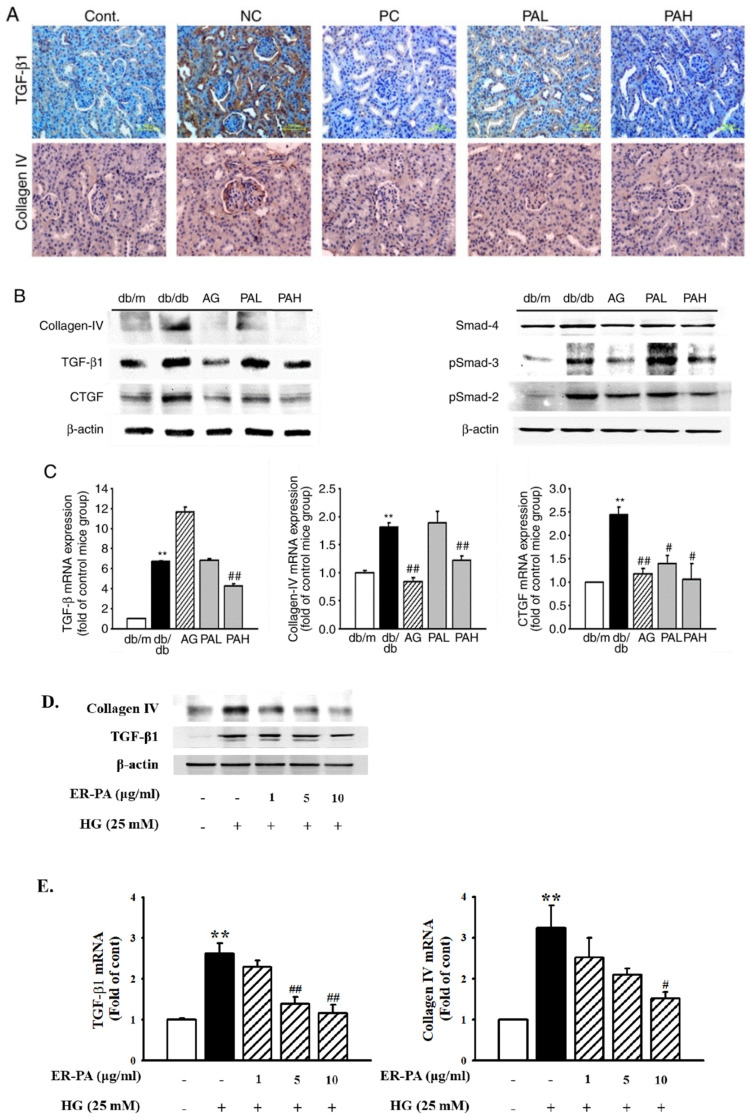
Effect of ER-PA on renal fibrosis. (**A**) Immunohistochemistry analysis showed that ER-PA therapy inhibits TGF-β1 and Collagen IV expression in the diabetic kidneys of db/db mice (*n* ≥ 3; magnification, ×200); (**B**) Expression of protein was determined with western blot analysis (*n* ≥ 3); (**C**) Expression of mRNA was determined by reverse transcription-quantitative PCR analysis (*n* ≥ 5). Values are expressed as mean ± standard deviation (*n* = 8). ** *p* < 0.01 vs. db/m; # *p* < 0.05, ## *p* < 0.01 vs. db/db; (**D**) Human renal mesangial cell lysates were used for western blot analysis with a primary antibody against TGF-β1 and collagen IV. β-actin was used as the internal standard in each sample; (**E**) Reverse transcription-quantitative PCR showing mRNA levels in ER-PA-treated and HG-stimulated mesangial cells. Each value represents the mean ± standard error of the mean of five independent experiments. ** *p* < 0.01 vs. db/m; # *p* < 0.05, ## *p* < 0.01 vs. HG alone. PA, *Polygoni avicularis*; HG, high glucose; AG, db/db mice daily treated with 20 mg/kg aminoguanidine; PAL, db/db mice daily treated with a low concentration (10 mg/kg) of ER-PA; PAH, db/db mice treated daily with a high concentration (50 mg/kg) of ER-PA.

The extent of glomerulosclerosis was examined by determining the expression of TGF-β1 and collagen IV, which are important regulators of ECM proteins. Immunohistochemistry results showed that TGF-β1 and collagen IV expression increased in the db/db group compared with that in the db/m group. However, treatment with AG, PAL, and PAH decreased TGF-β1 and collagen IV expression (Figure 6A). Western blotting and RT-qPCR demonstrated a significant increase in the expression of the factors related to TGF-β1/Smad signaling in the db/db group and their decreased expression in the AG and PAH group. Furthermore, CTGF protein and mRNA expression was inhibited by AG and PAH treatment (Figure 6B,C). To investigate the inhibitory effects of ER-PA on high glucose (HG)-induced renal fibrosis in human renal mesangial cells, western blotting and RT-qPCR was performed. The HG-induced TGF-β1 and collagen IV protein expression were significantly inhibited after treatment with ≥5 μg/mL ER-PA. RT-qPCR analysis showed that TGF-β1 and collagen IV mRNA expression was downregulated following ER-PA treatment (Figure 6D,E). These data showed that ER-PA can improve glomerular fibrosis associated with TGF-β/Smad and collagen accumulation in the diabetic nephropathy model.

### 3.6. Effect of the Ethanolic Extract from Rhizome of PA on Renal Inflammation

**Figure 7:**
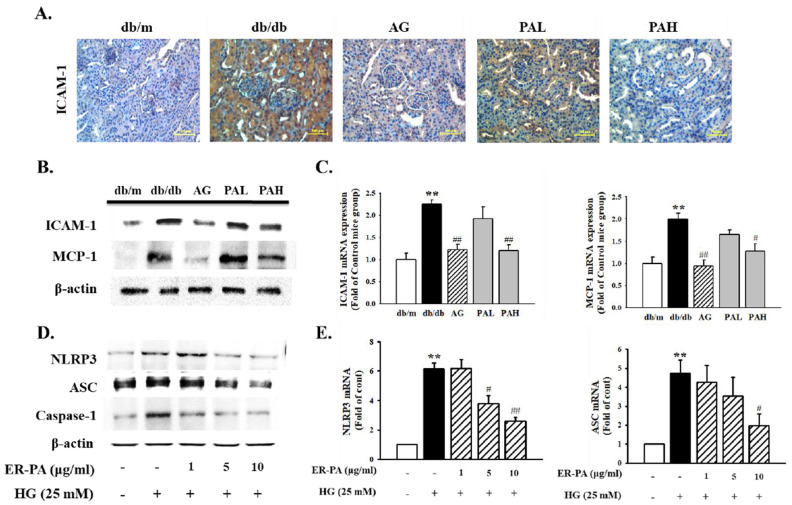
Effect of ER-PA on renal inflammation. (**A**) Immunohistochemistry analysis showed that ER-PA therapy inhibited ICAM-1 expression in the diabetic kidney of db/db mice (*n* ≥ 3; magnification, ×200); (**B**) Expression of protein was determined with western blot analysis (*n* ≥ 3); (**C**) Expression of mRNA was determined by reverse transcription-quantitative PCR (*n* ≥ 5). Values are expressed as mean ± standard error of the mean (*n* = 8). ** *p* < 0.01 vs. db/m; # *p* < 0.05, ## *p* < 0.01 vs. db/db; (**D**) Human renal mesangial cell lysates were used for western blot analysis with a primary antibody against NLRP3, ASC and caspase-1. β-actin was used as the internal standard in each sample; (**E**) Reverse transcription-quantitative PCR showing mRNA levels in ER-PA-treated and HG-stimulated mesangial cells. Each value represents mean ± standard error of the mean of five independent experiments. ** *p* < 0.01 vs. db/m; # *p* < 0.05, ## *p* < 0.01 vs. HG alone. PA, *Polygoni avicularis*; ICAM-1, intercellular adhesion molecule-1; MCP-1, monocyte chemoattractant protein-1; NLRP3, NLR family pyrin domain containing 3; ASC, apoptosis-associated speck-like protein; HG, high glucose; AG, db/db mice daily treated with 20 mg/kg aminoguanidine; PAL, db/db mice daily treated with a low concentration (10 mg/kg) of ER-PA; PAH, db/db mice treated daily with a high concentration (50 mg/kg) of ER-PA.

The extent of renal inflammation was examined by measuring the expression of inflammatory factors in db/db mice and human renal mesangial cells. The immunohistochemistry analysis showed that the expression of ICAM-1 increased in the db/db group compared with in the db/m group. However, administration of the AG, PAL, and PAH decreased ICAM-1 expression (Figure 7A). In addition, ICAM-1 protein and mRNA levels were significantly lower in the AG and PAH group (Figure 7B,C; *p* < 0.01). Further, the level of MCP-1 protein and mRNA significantly decreased in AG and PAH groups compared with in the db/db group (Figure 7B,C, *p* < 0.01; *p* < 0.05). To investigate the inhibitory effects of ER-PA on HG-induced renal inflammation in human renal mesangial cells, western blotting and RT-qPCR were performed. NLRP3, ASC and caspase-1 protein expression were significantly upregulated in HG-induced mesangial cells. However, NLRP3, ASC, and caspase-1 expression were significantly inhibited after treatment with ER-PA at ≥5 μg/mL. RT-qPCR indicated that HG treatment led to increased expression of NLRP3 and ASC mRNA. However, pretreatment of ER-PA significantly reduced the expression of NLRP3 and ASC mRNA in HG-induced renal mesangial cells (Figure 7D,E). These results indicated that ER-PA blocks the activation of NLRP3 inflammasomes and the expression of early inflammatory factors.

## 4. Discussion

The present study demonstrated the ER-PA-mediated amelioration of blood glucose levels and renal function parameters in db/db mice. The db/db mouse is an animal model of obesity-related diabetes and can be used to study kidney changes due to diabetes [33] The db/db mouse is overweight, hyperglycemic and hyperinsulinemic and exhibits increased kidney weight, glomerular mesangial matrix and albumin excretion [34] The present study showed that ER-PA significantly reduced the body weight and food/water intake of db/db mice. The urine volume in db/db mice was significantly higher than that in db/m mice and ER-PA treatment of these mice reduce the increase in urine volume compared with that in untreated db/db mice. There were obvious dysregulations in urinary albumin and plasma creatinine, which are considered as markers of renal function, along with histological changes in db/db mice. Although these levels were significantly higher in db/db mice compared with the db/m mice, the difference between untreated and ER-PA-treated db/m mice was small and the values remained within the normal range. Urinary albumin excretion may be a predictive factor for the prognosis of DN and urinary albumin indicates impairment of renal function [35,36]. It was verified that a reduction in urinary albumin level in type 2 diabetes is associated with reno-protective effects. In the present study, urinary albumin excretion significantly increased in db/db mice throughout the study period but decreased following ER-PA treatment. The glomeruli of db/db mice showed accelerated mesangial expansion, histologically characterized with an increase in the PAS-positive mesangial matrix area, as compared with that in the glomeruli of db/m mice. Treatment with ER-PA reduced the mesangial matrix expansion in db/db mice. These results suggested that ER-PA may improve renal function by reducing urinary albumin levels in a diabetic animal model. ER-PA inhibits several symptoms of diabetes, depending on the glucose index. Insulin resistance is a major finding in patients with type 2 diabetes mellitus. Insulin resistance may exist even earlier in patients with mild renal disease (insulin resistance assessed with OGTT or HOMA-IR) and DN [2,37]. The HOMA-IR index is the most widely used index, which represents the product of glucose and insulin concentrations divided by a factor [38]. Plasma insulin and HOMA-IR are markedly elevated in db/db mice compared with db/m mice [39]. In the present study, plasma glucose and insulin concentrations were significantly higher in db/db mice compared with the untreated db/m mice but decreased following ER-PA treatment. These results suggested that ER-PA ameliorates insulin resistance by improving blood glucose and insulin concentrations under diabetic conditions. The present study found that ER-PA improved early glomerular damage by recovering the loss of glomerular nephrin expression. However, immunochemical staining results confirm that nephrin expression was stained not only in Bowman’s pockets, but also in the glomeruli. Nephrins should be expressed in most of the glomeruli, but the present study did not yield such results. However, as with other studies, there were cases in which no noticeable manifestations were observed within the glomerulus [40,41]. Therefore, ER-PA may prove to be effective for the treatment of renal dysfunction. DN is a morbid microvascular complication associated with diabetes and is the most common cause of end-stage renal disease [42]. In DN, the accumulation of ECM components in the glomerular mesangium and tubulointerstitium causes early glomerular hypertrophy and eventually glomerulosclerosis and tubulointerstitial fibrosis [43]. The concentration of collagen IV increases with DN progression in patients and db/db mice [44]. Collagen IV accumulation is a crucial phenomenon underlying mesangial expansion [45]. The mesangial expansion and glomerular fibrosis observed in db/db mice may result from molecular changes within the renal tissue, including the activation of various pro-inflammatory cytokines and growth factors [46]. TGF-β1 is another important factor in the pathogenesis of DN and mediates an inflammatory response, which aggravates ECM secretion involving fibronectin and collagen accumulation and accelerates glomerulosclerosis in diabetes [47]. To further clarify the effect of ER-PA on glomerular fibrosis, immunohistological staining for collagen IV and TGF-β1 in glomeruli was performed. Collagen IV and TGF-β1 expression was suppressed by ER-PA, compared with untreated db/db mice. ER-PA decreased TGF-β/Smad-2 protein and mRNA expression in the kidneys of db/db mice, as confirmed with western blotting and RT-qPCR, respectively. Furthermore, the expression of Smad-2, Smad-3, and Smad-4 markedly decreased in the nuclear fraction derived from diabetic mouse kidneys following ER-PA treatment. Fibronectin and collagen IV levels in ER-PA-treated db/db mice were lower compared with those in untreated db/db mice. Therefore, ER-PA suppressed renal fibrosis that was activated in the kidney of db/db mice, disturbed TGF-β/Smad activity and promoted ECM degradation. In recent years, clinical and experimental evidence has indicated the important role of inflammatory cytokines in the development and progression of DN [48]. ER-PA ameliorated renal inflammation through the suppression of inflammatory factors in diabetic db/db mice, emphasizing its renoprotective effect in the DN accelerated by renal fibrosis and inflammation in type 2 diabetic db/db mice.

DN is characterized by glomerular alterations in the renal tissue, including thickening of the glomerular basement membrane and mesangial matrix expansion, leading to the evolution of glomerulosclerosis [49]. Increased mesangial cell proliferation as well as the accumulation of ECM components such as collagen in the glomeruli are some of the characteristic pathologic features of early stage DN [50]. Renal inflammation and subsequent fibrosis are critical processes leading to end-stage DN [51,52]. The NLRP3 inflammasome is a mediator of inflammation and contributes to the progression of chronic kidney disease. Its activation is linked to autoinflammatory diseases [17]. The present study confirmed the effect of ER-PA on inflammatory and fibrotic changes in primary human renal mesangial cells induced by the HG. As with the in vivo studies, these results showed that ER-PA markedly ameliorated HG-induced mesangial fibrosis in renal glomerular mesangial cells through the downregulation of the TGF β signaling pathway. Furthermore, ER-PA inhibited HG stimulation and increased the expression of inflammation-related factors, including ICAM-1, MCP-1 and NLRP3 inflammasome. Therefore, these findings demonstrated the significant protective effect of ER-PA against diabetic renal injury via the control of TGF-β signaling pathways and inflammatory factors leading to DN. However, the present study had several limitations. Aminoguanidine used in the present study is a representative AGE inhibitor, but it is not a clear positive control for diabetic nephropathy improvement. Therefore, further studies taking this into account are needed for a clearer identification of the improvement of diabetic nephropathy.

## 5. Conclusions

In conclusion, the present study demonstrated ER-PA resulted in significant ameliorate of urinary albumin excretion, oral glucose tolerance, and insulin resistance index. Moreover, the beneficial effects of ER-PA on DN that are mediated through the alleviation of glomerular fibrosis in the kidneys induced by diabetes as well as amelioration of inflammation. Taken together, the function of ER-PA against diabetes-associated renal dysfunction may provide new insights into the development of therapeutic drugs for DN. Moreover, further research to elucidate detailed mechanism of ER-PA at the cellular and molecular levels in diabetic nephropathy needs to be done.

## Data Availability

Data available on request from the authors.
